# Hydrothermally reinforcing hydroxyaptatite and bioactive glass on carbon nanofiber scafold for bone tissue engineering

**DOI:** 10.3389/fbioe.2023.1170097

**Published:** 2023-05-24

**Authors:** Asmaa M. Abd El-Aziz, Eman Serag, Marwa Y. Kenawy, Azza El-Maghraby, Sherif H. Kandil

**Affiliations:** ^1^ Fabrication Technology Research Department, Advanced Technology and New Materials Research Institute (ATNMRI), City of Scientific Research and Technological Applications (SRTA-City), Alexandria, Egypt; ^2^ Marine Pollution Department, Environmental Division, National Institute of Oceanography and Fisheries, Alexandria, Egypt; ^3^ Department of Materials Science, Institute of Graduate Studies and Research, Alexandria University, Alexandria, Egypt

**Keywords:** carbon nanofibers, bioceramics, bioglasses, cytotoxicity, osteogenesis

## Abstract

As a bone tissue engineering scaffold, the objective of this study was to design hierarchical bioceramics based on an electrospun composite of carbon nanofibers (CNF) reinforced with hydroxyapatite (HA) and bioactive glasses (BGs) nanoparticles. The performance of the nanofiber as a scaffold for bone tissue engineering was enhanced by reinforcing it with hydroxyapatite and bioactive glass nanoparticles through a hydrothermal process. The influence of HA and BGs on the morphology and biological properties of carbon nanofibers was examined. The prepared materials were evaluated for cytotoxicity *in vitro* using the water-soluble tetrazolium salt assay (WST-assay) on Osteoblast-like (MG-63) cells, and oste-ocalcin (OCN), alkaline phosphatase (ALP) activity, total calcium, total protein, and tar-trate-resistant acid phosphatase (TRAcP) were measured. The WST-1, OCN, TRAcP, total calcium, total protein, and ALP activity tests demonstrated that scaffolds reinforced with HA and BGs had excellent *in vitro* biocompatibility (cell viability and proliferation) and were suitable for repairing damaged bone by stimulating bioactivity and biomarkers of bone cell formation.

## 1 Introduction

Tissue engineering and regenerative medicine seek to repair damaged tissues, bone defects caused by severe fractures, and bone tumours by providing cells with the resources they require (Aoki et al., 2020). There are several methods for treating bone defects, including bone grafting (Dimitriou et al., 2011), bone transplantation (Yang et al., 2018), and the membrane technique (Taylor et al., 2012). Instead of traditional passive implants, active living biomaterials and structures are being used (Aoki et al., 2020). An increasing number of geometries, materials, and technologies have been researched and developed over the last few decades in order to create numerous artificial cell tissues, niches, and extracellular matrices. The ideal material for bone tissue regeneration should, in general, have excellent biocompatibility, biodegradability with controllable degradation kinetics, ease of production, and adequate mechanical properties. To counteract the current biomechanical inconsistencies, size-related constraints, and biocompatibility problems, manufacturing technologies with new material families and processing methods should be researched (Islam et al., 2022).

Nanofibers are a potentially useful material for tissue regeneration because they can adapt to cell adhesion and proliferation. Several nanofiber materials, such as natural and synthetic polymers and their composites, are used to grow new bone and fill in holes in bone (Aoki et al., 2009; Nareswari et al., 2022).

Due to the fact that carbon nanofibers express the same structure and mechanical properties as the natural extracellular matrix, they possess remarkable electrical activity, intriguing biocompatibility, and high stability after implantation. It has been demonstrated that carbon fiber for example (Demirkiran et al., 2010; Kim et al., 2021) synthesized a silk fibroin/carbon nanofiber scaffold with a unique electrical conductivity that allows it to adapt to the piezoelectric properties of bone in order to induce the osteogenic differentiation of Bone marrow mesenchymal stem cells (MSCs) cultured on a three-dimensional scaffold.

Alternatively, the addition of other chemicals to CNF may provide additional benefits. Hydroxyapatite (HAp) is a bioceramic material that the primary constituent of bone tissue. Ca^2+^ release by HAp can govern the osteogenic differentiation of stem cells. Increased extracellular Ca^2+^ concentrations lead to a significant rise in intracellular Ca^2+^ concentrations. In turn, intracellular Ca^2+^ can activate signalling pathways, such as those induced by calcium/calmodulin-dependent protein kinase, which are essential for various facets of osteoblast growth and differentiation (Sun et al., 2022).

It can also bind to some parts of bone through an ion exchange reaction that makes apatite carbonate (Rizwan et al., 2021). Furthermore, it can also be broken down or taken back up by the cells’ metabolism. Most of the time, implants are put into its surface, channels, or canals to make bones (Seo and Lee, 2009; Fu et al., 2019). In addition, it is responsible for Osteoinduction is the process of getting immature cells to change into mature osteoblasts. It also involves bone adhesion, cell growth, and differentiation of osteoblasts, which slowly replace living bones (Rizwan et al., 2021). On the other hand, HAP is incorporated into biopolymers to improve mechanical strength and biocompatibility (Feng et al., 2022).

Bioactive glasses are very interesting materials for the engineering of bone and soft tissue (Rizwan et al., 2021). In addition to aiding angiogenesis and osteogensis processes, they exhibit a unique capacity for bone-bonding through the formation of HA layer. Moreover, they induce an anti-inflammatory response and possess antibacterial properties, giving them great potential for bone regeneration and wound healing (Pantulap et al., 2021).

The incorporation of BGs as hard fillers into nanofibers improves mechanical properties, induces mineralization, and stimulates osteogenesis (Zheng et al., 2021). Wang and colleagues developed a beta cyclodextrine modified mesoporous bioactive glass nanoparticles/silk fibron hybrid nanofiber for the treatment of osteoporosis. The incorporation of BGsNPs into the fiber increased *in vitro* apatite formation and osteoblast, alkaline phosphatase activity, and cell proliferation in comparison to silk fiber alone (Wang et al., 2018). Previous attempts to combine bioglass with other materials as a scaffold to enhance biological performance and accelerate osteogenic properties, (Su, et al., 2022), developed ternary structured composite scaffolds via 3D printing technique to combine bioglass with biphasic calcium phosphate (BCP) which lead to progress the mechanical strength, bioactivity, degradability, and biocompatibility of the composite ceramic scaffolds.

Here, we present a straightforward, economically viable strategy for depositing HA and BGs into CNF to produce synthetic scaffold rich in apatite, a mineral similar to that found in bone.

In this research, we used a hydrothermal approach to nucleate HA and BGS on CNF’s surface via an ion dissociation route. A prior report by the authors not only suggested that hydrothermal Molecular transformation into a thermodynamically favored crystalline stable a-phase was induced by treatment, which also aided in the nucleation and assembly of inorganic compounds from surrounding metal ions onto fiber substrates (Abdal-hay et al., 2021).

In our previous research, the carbonization of CNF/HA nanocomposite was carried out using two methods: modification during electrospinning and hydrothermal modification after carbonization. We revealed that hydrothermally prepared nanofibers have a higher proportion of HA, which enhances surface hydrophilicity and induces osteogenic features (Abd El-Aziz et al., 2021b). On the other hand, Feng et al. (2022) used a hydrothermal method to deposit HA nanoparticles on the surface of carbon nanotubes and add them to a poly (-caprolactone) (PCL) scaffold.

**SCHEME 1:**
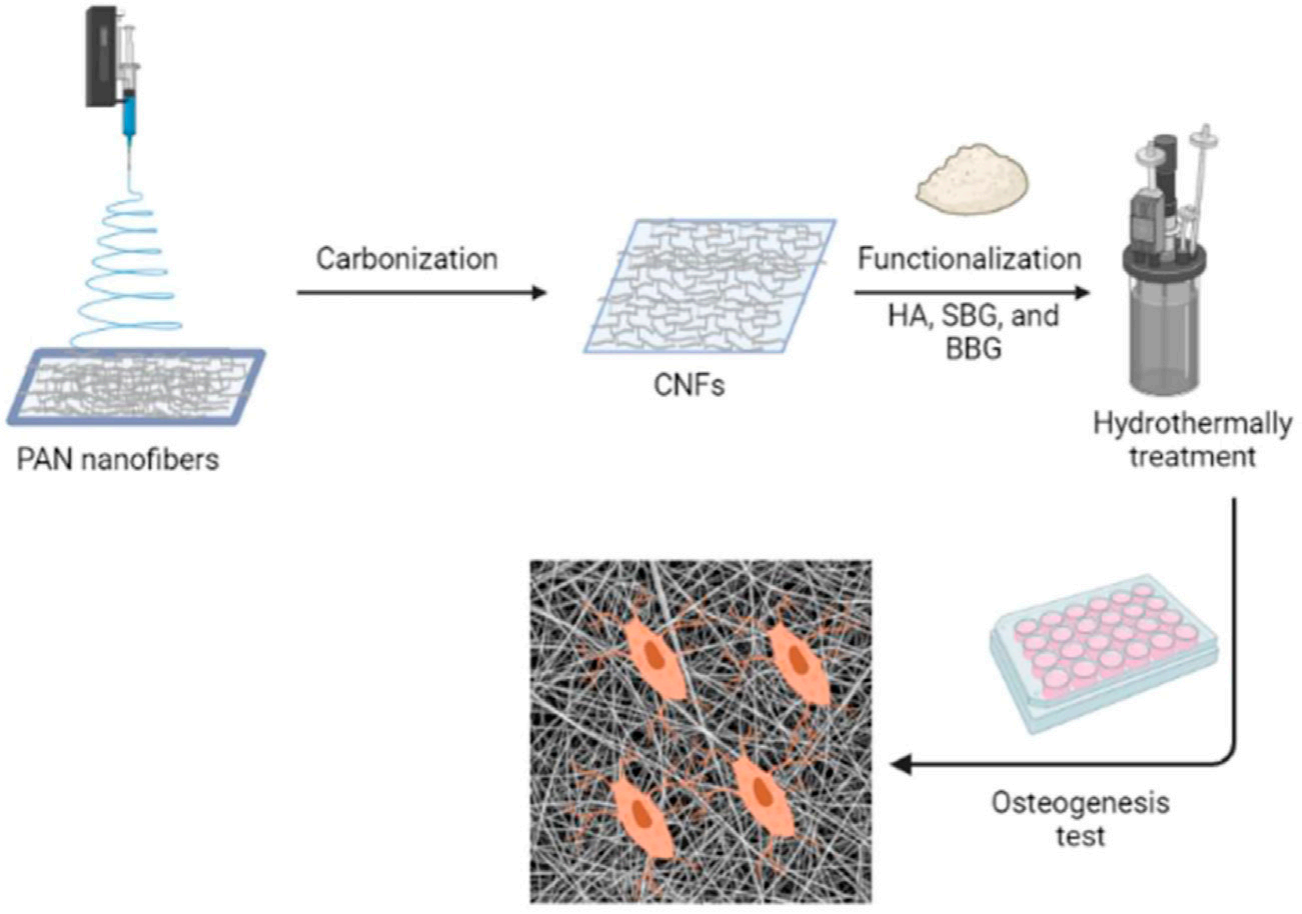
Graphical abstract representing the synthesis of CNFs and their potential for osteogenesis.

To the best of our knowledge, this is the first time that HA and bioglass have been combined to increase the bioactivity of a carbon nanofiber scaffold using hydrothermal technique. Scanning electron microscope (SEM), transmission electron microscope (TEM), Fourier transform infrared (FTIR), X-ray diffraction (XRD) and the elemental analysis were used to characterize the physicochemical properties and morphologies of the nanofiber scaffold. In addition, the cytocompatibility of MG-63 cells was evaluated by analyzing cell adhesion and proliferation as shown in Scheme 1.

## 2 Materials and methods

### 2.1 Materials

Polyacrylonitrile (PAN) (average molecular weight = 150,000 g/mol, d = 1.184 g/mL at 25°C) and N,N dimethylformamide (C_3_H_7_NO) were purchased from Sigma-Aldrich (Sigma-Aldrich, St. Louis, MO, United States). Naturally prepared hydroxyapatite, silicate and borosilicate bioactive glasses were prepared at our labs according to our previous work (Abd El-Aziz et al., 2017). Dulbecco’s modified Eagle’s medium, with 4.5 g/L glucose and L–glutamine (DMEM), Penicillin 10,000 IU/mL and streptomycin 10,000 μg/mL, and Trypsin-EDTA solution containing 0.25% trypsin was manufactured by Lonza (Verviers, Belgium). Fetal bovine serum (FBS, heat inactivated) was purchased from EuroClone SpA (Milan, Italy). 2-(4-iodophenyl)-3-(4-nitrophenyl)-5-(2, 4-disulfophenyl)-2H tetrazolium, monosodium salt (WST-1) assay kit (Cell Proliferation Reagent) (ab 155902) supplied by Abcam (Wal-tham, MA, United States). Human MG-63 cell line and Human Skin Fibroblas (HSF) were obtained from Nawah Scientific Inc., (Cairo, Egypt).

### 2.2 Methods

#### 2.2.1 Synthesis of carbon nanofibers

The polymeric nanofibers were prepared using PAN solution (5 wt%). The polymeric solution was fed at a constant rate of 0.5 mL.h-1 into a syringe needle using a syringe pump. By applying a specified voltage of 20 kV, PAN solutions were electrospun on a flat collector with an aluminium foil that was located at a distance of 10 cm from the needle (Abd El-Aziz et al., 2017). To create carbon nanofibrous membranes, the stabilised and carbonised polymeric nanofibrous membranes underwent thermal processing. The stabilisation involves heating the polymeric nanofibers in two steps; the dehydrogenation and the cyclization, but the carbonization process involves incubating the stabilised nanofibers at high temperature in an inert gas (Ar) to expel all residual gases except C and H (Rajzer et al., 2012). The stabilisation process was done by heating the prepared polymeric nanofibers (80–150 μm in thickness) at a rate of 1.0°C/min up to 250°C, then incubating the fibres at 250°C for 2 h in an air atmosphere. The second step was the carbonization, which took place by heating the stabilised nanofibers at a rate of 5°C/min and incubating them at 1,000°C for 1 h under Ar gas (Abd El-Aziz et al., 2017; Aziz A et al., 2017; Abd El-Aziz et al., 2021b).

#### 2.2.2 Hydrothermal functionalization of CNFs with bioceramics and bioglasses

The naturally annealed HA and the prepared bioactive glasses (silicate and borosilicate) (SBG and BBG), which have been prepared at our labs (Abd El-Aziz et al., 2017; Abd El-Aziz et al., 2021a), were dispersed in distilled water with a concentration of 8 wt%. Briefly, HA has been prepared from bovine bone after pretreatment, drying, then grinding, sieving, finally calcination at 700°C for 6 h, to remove all organic materials, as shown in this article (Abd El-Aziz et al., 2017). SBG and BBG have been prepared by sol-gel technique, SBG-NPs, 70% SiO_2_, 24% CaO, 6% P_2_O_5_, while BBG-NPs, with a mole composition of 55% SiO_2_, 24% CaO, 6% P_2_O_5_, and 15% B_2_O_3_. TEOS were added as a precursor to silicon dioxide (SiO_2_), and 2 M nitric acid (as a hydrolysis catalyst). Next, TEP as a precursor to phosphorus penta-oxide (P2O5) was added and then Ca (NO_3_)_2_·4H_2_O (11.214 g) was added as a precursor of calcium oxide (CaO).

Finally, boric acid H_3_BO_3_ (6.9 g) was added as a precursor of (B_2_O_3_) in case the preparation of BBG particles. At the end of the reaction for both glasses, ammonium solution (a gelation catalyst) was added dropwise to each solution while vigorously stirring. The resulting gels were dried, then sintering took place at 700°C for 2 h.

The prepared CNF membranes (30 × 20 mm^2^) were immersed in the as-obtained suspension or solution (40 mL), immediately placed into a stainless-steel autoclave system, and hydrothermally treated for 90 min at 130°C. The resulting samples were left to dry at room temperature and were then placed in a vacuum oven (10 mbar) at 40°C for 72 h prior to further processing.

#### 2.2.3 Characterization of nanofibers mats

The morphology of nanofibrous mats was investigated using a scanning electron microscope (SEM) (JEOL JSM 6360LA, Japan). The average nanofiber diameters were measured using the image J software with randomly selected SEM micrographs. The nanofibrous mats were characterised by a high-resolution transmission electron microscope (JEOL 2100 PLUS, Japan). Transmission electron microscopy was used for the purposes of imaging, crystal structure revelation, and elemental analysis (“qualitative and semi-quantitative”) analysis. Two imaging modes were used: bright field at 200 kV electron accelerating voltage using a lanthanum hexaboride (LaB6) electron source gun and diffraction pattern imaging. An Eagle CCD camera with (4 k ∗ 4 k) image resolution was used to acquire and collect transmitted electron images. By using EDS mapping analysis, which is performed through a unit with TEM microscope, the elemental analysis of the produced nanofibrous mats has been investigated. The chemical structures of CNF and CNF with both (bioactive glass and hydroxyapatite) nanofibers were studied by FTIR spectroscopy. The samples were mulled with dry potassium bromide (KBr) powders at room temperature. The spectrum is recorded in the wavenumber range of (4,000–350) cm-1. At room temperature, X-ray diffraction scans of nanofiber samples were obtained using the X-ray 7,000 Shimadzu-Japan. By measuring the Bragg angle (2θ) in the range of 10–80 degrees. The X-ray source is a Cu target with a scan speed of 4 degrees per minute and a voltage of 30 KV and 30 Ma.

#### 2.2.4 Cell Viability and Proliferation Test

Prior to biological experiments, the samples (circular disc with a thickness of 5.0 mm) were sterilised with 70% ethanol for 30 min, washed three times with sterile PBS, plated in a 96-well cell culture plate, and immersed in culture medium (50 μL of DMEM media supplemented with 100 mg/mL streptomycin, 100 units/mL penicillin, and 10% heat-inactivated foetal bovine serum). Subsequently, the culture medium was removed and 50 μL fresh culture medium containing (3 × 10^3^) of both MG-63 cells and HSF was added. For 24 h, the cells were incubated in a CO_2_ incubator (37°C, 5% CO_2_, and 90% relative humidity). At the end of the treatment period, 10 μL WST-1 reagent was added, and the absorbance was measured after incubating for 1 h with the WST-1 reagent at ʎ = 450 nm using a BMGLABTECH®-FLUO star Omega microplate reader (Allmendgrün, Ortenberg) (Sharma et al., 2014; Alaufi et al., 2017).

The % viability was calculated as the following:

ATAC×100 (1)

AT = mean absorbance of cells treated with different CNF samples.AC = mean absorbance of control untreated cells with cultures.

#### 2.2.5 Evaluation of biological and osteogenic differentiation markers

Human MG-63 cells at a concentration of 1 × 10^5^ cells/well were allowed to grow in culture media for 2 days to reach 70% confluence, then collected. The prepared scaffolds (circular disc with a thickness of 5.0 mm) were sterilised as before, but they were plated in 24-well cell culture plates with 500 μL of culture medium (CM). For 3 days, MG-63 cells were incubated with scaffolds at 37°C with 5% CO_2_. The adherent cells were enzymatically detached with trypsin/EDTA solution (1×) and then the suspended cells were collected by centrifugation at 400 × g for 5 min. The cell pellets were washed three times with phosphate buffered saline (PBS). The supernatant was collected for quantification of biochemical parameters.

#### 2.2.6 Biochemical and osteogenic differentiation markers:

Bone proliferation markers such as osteocalcin (OCN), alkaline phosphatase (ALP), and intracellular calcium concentration were assayed using commercial kits supplied by Bioneovan Co., Ltd. (Beijing, China), and Genesis Lab (Cairo, Egypt), respectively. While total protein concentration was assayed according to BioMED Diagnostics (Hannovar, Germany), and tartrate-resistant acid phosphatase (TRAcP) as a marker of bone resorption was assayed according to Bioneovan Co., Ltd. (Beijing, China). Calcium has been measured according to the kit “Calcium oCPC”, while the Calcium in an alkaline medium combines with o-Cresolphthalein Complexone to form a purple coloured complex. Intensity of the formed colour is directly proportional to the amount of calcium present inside the cells.

#### 2.2.7 Statistical Analysis

The Statistical Product and Service Solutions (SPSS) software package, version 20.0 (IBM Corp., Armonk, NY, United States), was used for data analysis. The mean and standard deviation are used to represent data. Significance among samples at *p* < 0.05 was assessed by using one-way analysis of variance (ANOVA).

## 3 Results and discussion

### 3.1 The morphology and the physical characterization of the CNFs composites scaffolds

**FIGURE 1:**
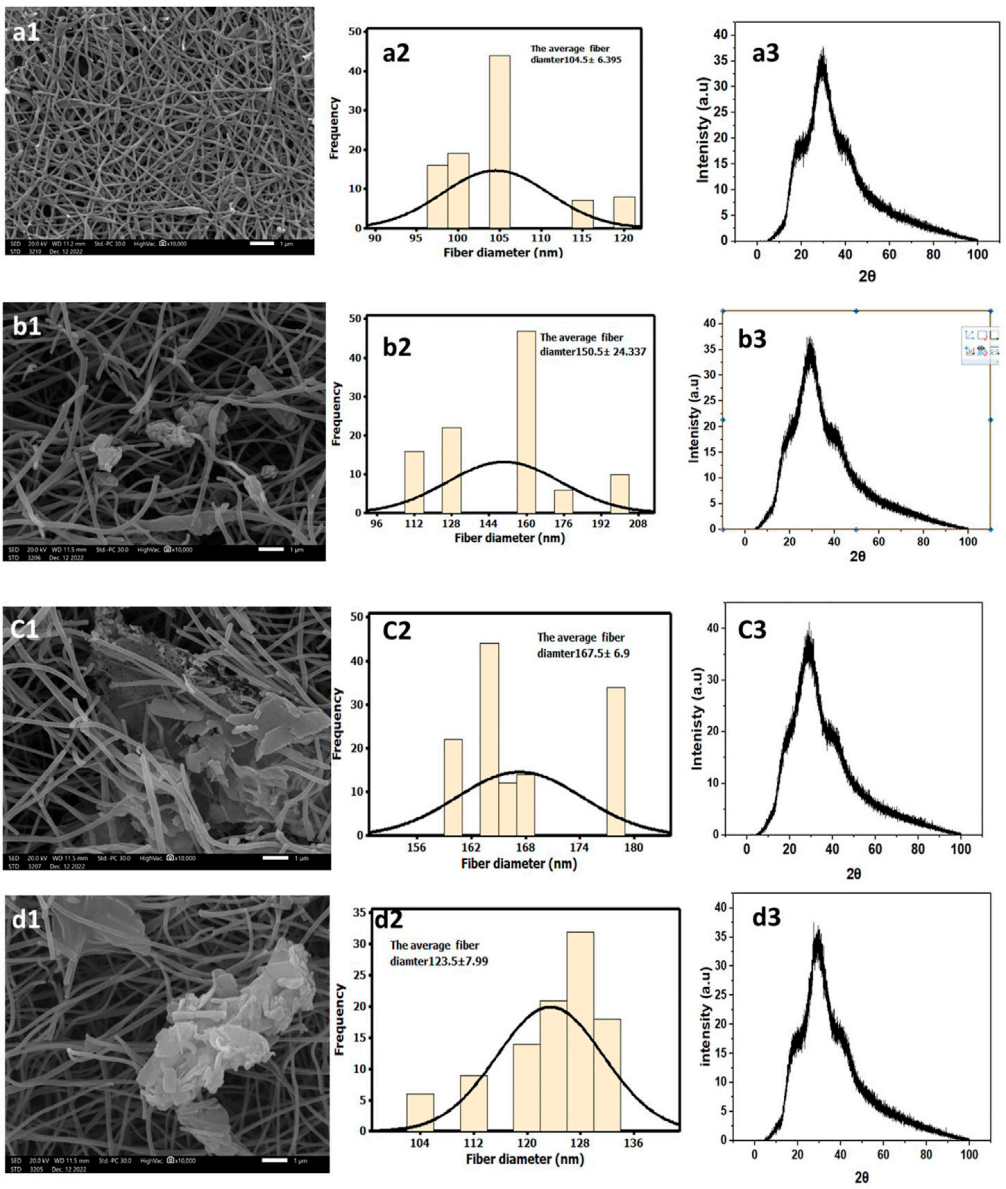
Characterization of CNF, and their composites. Groub a for CNF, group b for CNF-HA, group c for CNF-SBG, and CNF-BBG. SEM at **(A1–D1)** and fiber diamters at **(A2–D2)** while XRD anaylsis at **(A3–D3)**.

**FIGURE 2:**
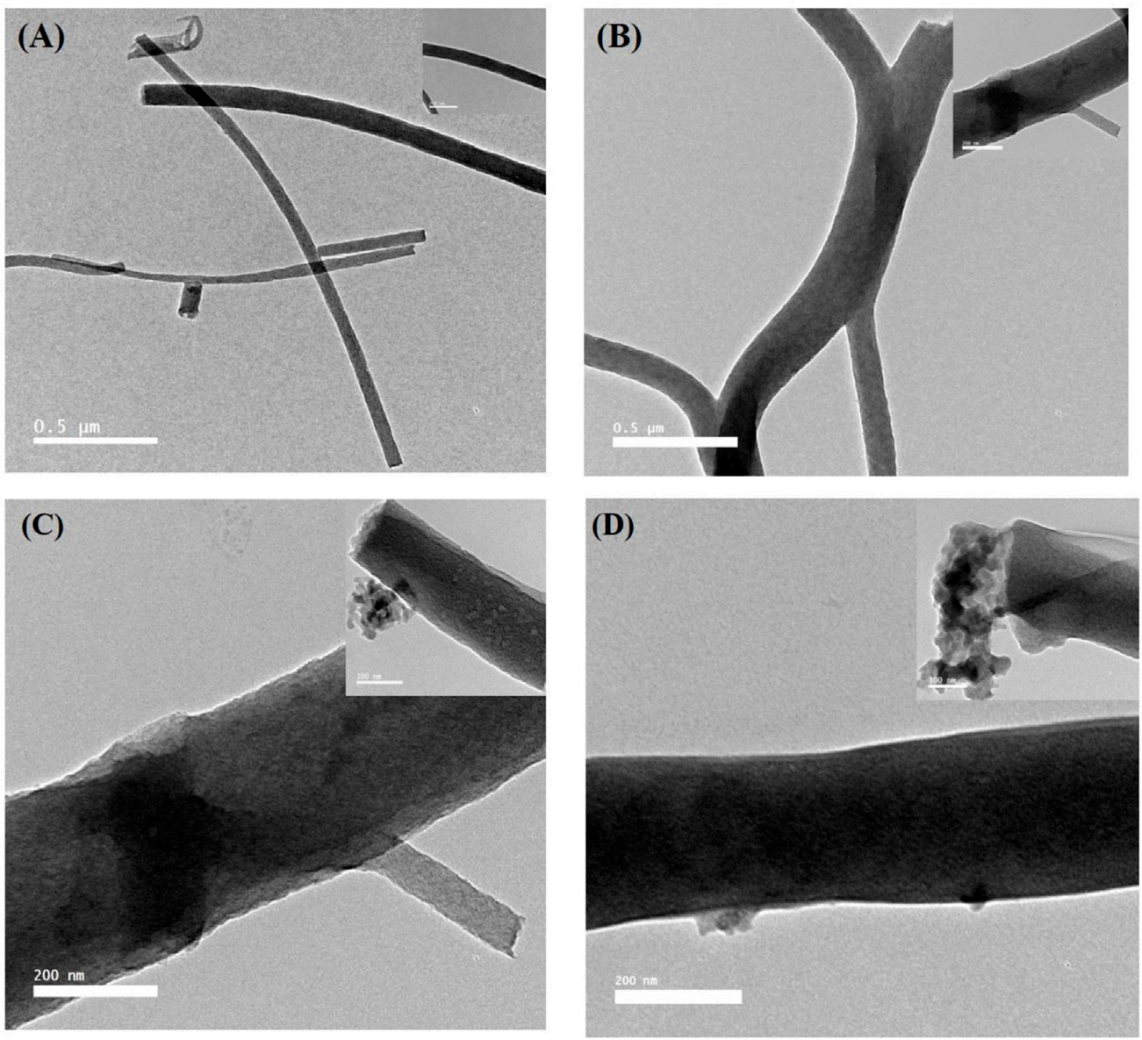
TEM images of the CNF, CNF-HA, CNF-SBG, and CNF-BBG nanofibrous mat, respectively. High magnified images on the top - right of each image.

The SEM morphology of CNF, CNF-HA, CNF-SBG, and CNF-BBG is presented in Figure 1. The pure CNF mats were smooth and homogenous, with an average diameter of 104.5 ± 6.395 nm. The successful incorporation of HA was seen in the form of aggregation mass inside the fibre in the case of CNF-HA, and the average diameter was 150.5 ± 24.337 nm. The addition of HA also makes the surface of the fibre rougher, and this result is consistent with those of previous work (Hartatiek et al., 2021). It should be remembered that the embedded HA produces a rough nanofiber surface, which is expected to increase surface wettability and encourage the attachment of bone cells and the formation of new bone differentiation (Bodhak et al., 2009; Hartatiek et al., 2021). Additionally, for CNF-SBG and CNF-BBG, respectively, the fiber diameter increased to (167.5 ± 6.9) nm and (123.5 ± 7.99) nm after silicate and borosilicate bioactive glass doping. And this indicates that SBG and BBG were successfully added to the fiber, and the outcome was consistent with earlier research (Boschetto et al., 2020). Figures 1A3–D3 show the XRD patterns of all samples. There were two peaks that were identified as (002) and (101) corresponding to carbon graphitization that revealed CNF synthesis, but there were none for HA, SBG, or BBG. This result is consistent with previous work (Yang et al., 2013; Aziz A et al., 2017). Figure 2 shows the transmission electron microscopy (TEM) images of the prepared samples that refer to SBG, BBG, and HA nanoparticles in the CNF, where they appear as a rough surface of carbon nanofiber. The CNF is depicted in the images as a nanofibrous web, after incorporation of HA, SBG, and BBG the smaller nanoparticles being located within the nanofibers and the larger ones being partially captured on their surfaces.

**FIGURE 3:**
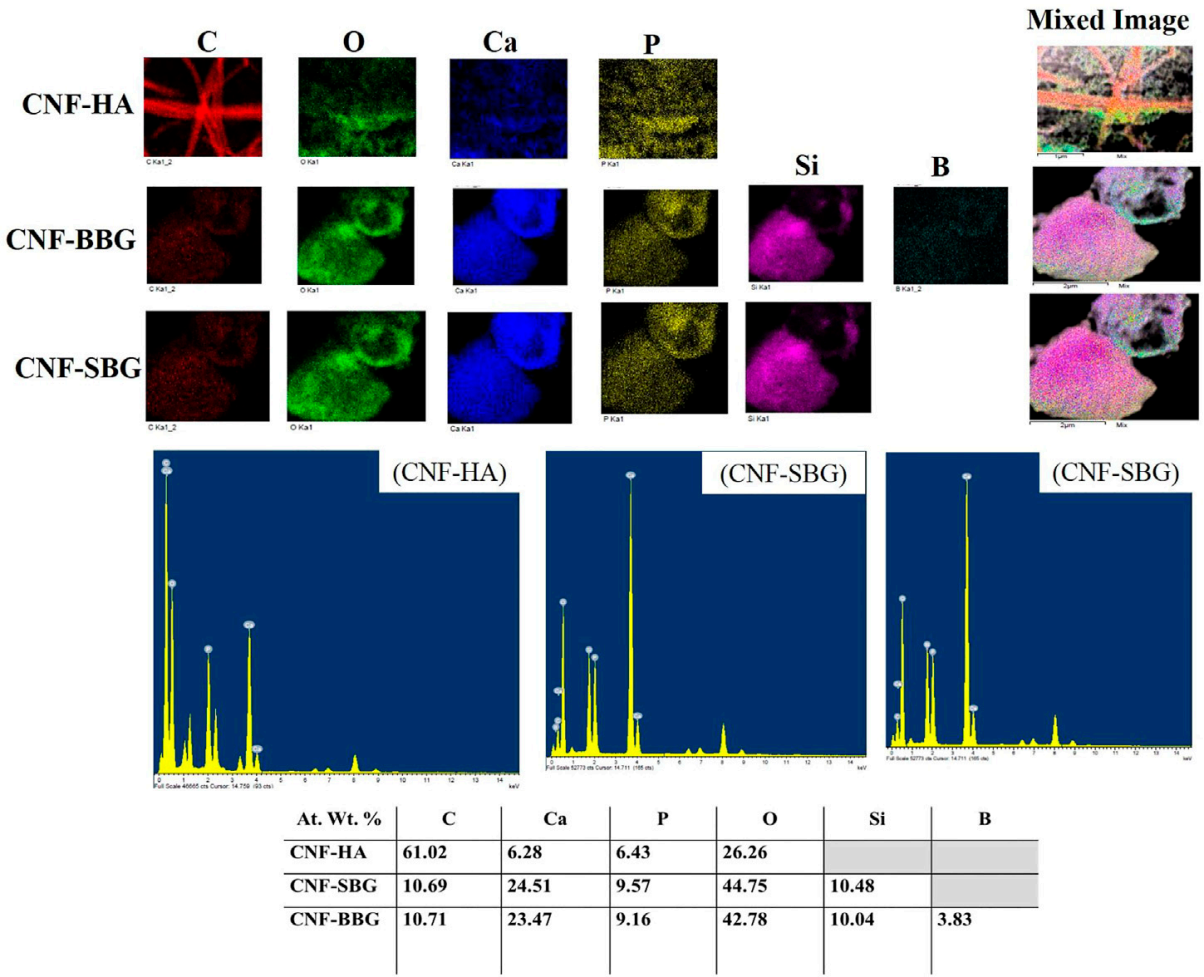
EDS mapping of the CNF-HA, CNF-SBG, and CNF-BBG nanofibrous mat with their elemental analysis.

Furthermore, the composite carbon nanofiberous mats were assessed using EDs mapping to confirm the existence of hydroxyapatite and bioactive glass particles in the fibres (Figure 3). The presence of elements found in hydroxyapatite, such as Ca and P, and bioactive glasses, such as Si, Ca, P, and B in the case of BBG nanoparticles, confirmed the incorporation of ceramic and glass particles in the nanofiberous matrix, despite the fact that EDX analysis is not a quantitative study. The ratios of each element present in the fibrous carpets were calculated using the EDX elemental analysis. Observed CNF had an 85% carbon content, which is reasonably consistent with expectations based on literature for PAN carbonization (Kretzschmar et al., 2020). The carbon content of CNF decreased by reinforcing HA and biglasses, and this result is similar to our earlier work (Aziz A et al., 2017).

**FIGURE 4:**
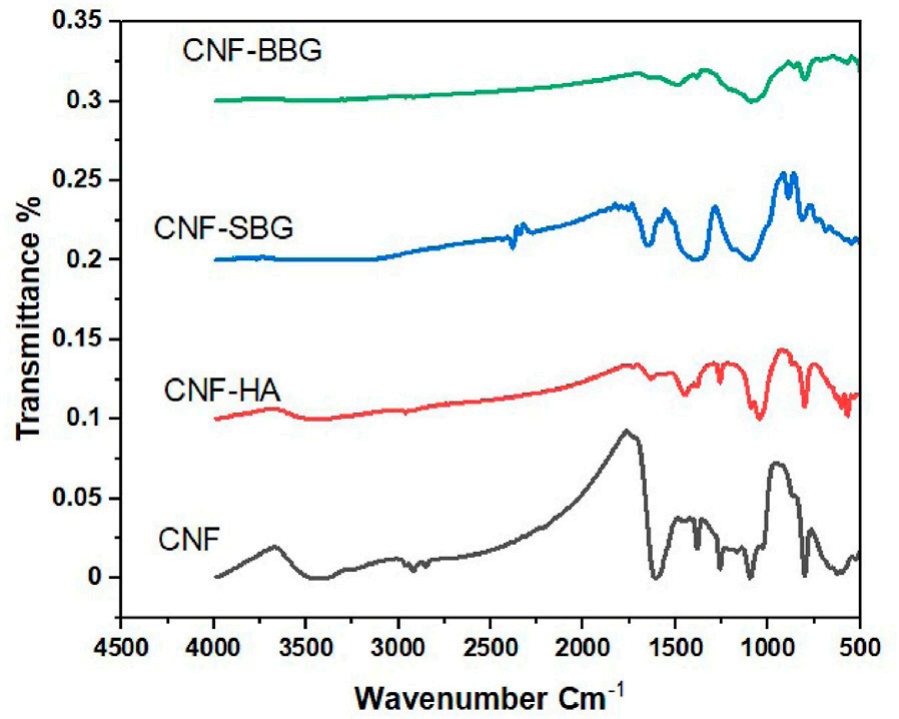
FTIR spectra for CNF, CNF-HA, CNF-SBG, and CNF-BBG.

The possible interactions between CNF and HA, SBG, and BBG were investigated using FTIR spectra (Figure 4). CNF’s FTIR spectra revealed two distinct peaks at 1,218 cm^−1^ and 1,566 cm^−1^, as well as small peaks at 1961 cm^−1^, 2033 cm^−1^, and 2,222 cm^−1^ (Ibupoto et al., 2018). The spectra at 1,218 cm^−1^ are related to the C-H bond and show the formation of a heteroaromatic ring with a double bond, whereas the spectra at 1,580 cm^−1^ show C=C stretching vibration in the aromatic ring of CNF (Abdullah et al., 2018; Senthil Kumar et al., 2019). The C=C bond is represented by the small peaks at 1961 cm^−1^, 2033 cm^−1^, and 2,222 cm^−1^. Following HA reinforcement, peaks appear in the spectra at 1,030 cm^−1^, 870 cm^−1^, and 3,400 cm^−1^ corresponding to PO_4_^3−^, CO_3_^2−^, and OH of HA, respectively (Manoj et al., 2015; Hartatiek et al., 2021). The CNF-SBG spectra demonstrate the silicate bioactive glass SBG’s distinctive absorption bands. At 800 cm−^1^, the Si-O vibration is assigned the week bond (Lao et al., 2017). The stretching mode of the symmetric Si-O-Si bridge was responsible for the absorption band at 970 cm^−1^, while the asymmetric vibration band was visible at 1,250 cm^−1^ (Szewczyk et al., 2021). The spectra of CNF-BBG show typical borosilicate glass peaks, with vibrations at 686 and 1,450 cm^−1^ attributed to B-O-B stretching and 900 cm^−1^ attributed to B-O starching and for the BO_4_ group (Ciżman et al., 2020).

### 3.2 Biocompatibility and cytotoxicity properties of CNFs composites scaffold

**FIGURE 5:**
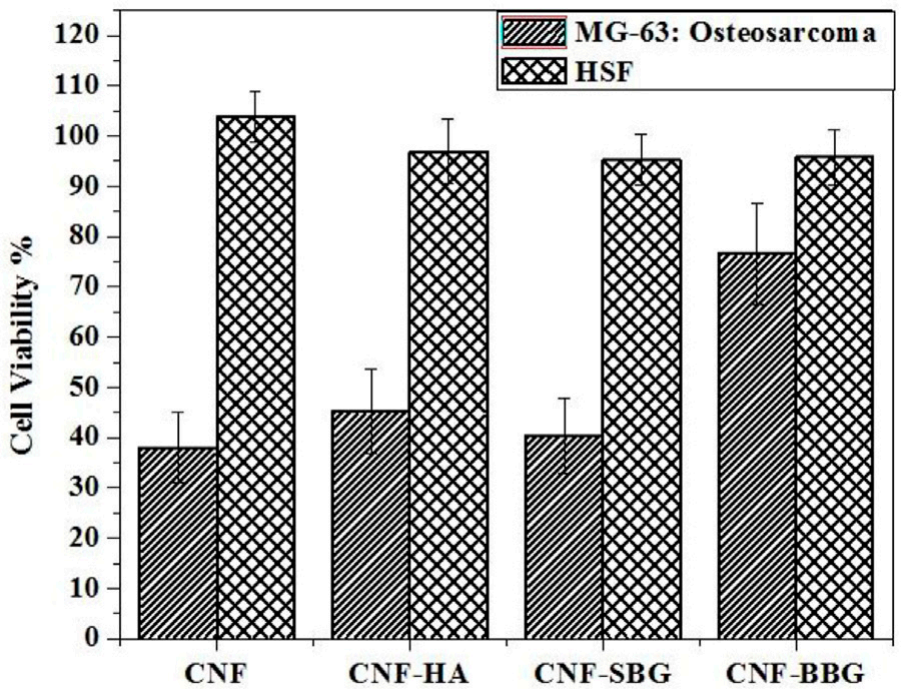
Cell viability of all groups with both (MG63 and HSF).

Figure 5 presents the quantitatively examined degrees of cell proliferation on the prepared scaffolds through the WST-1 assay method on human skin fibroblasts (HSF) as normal cells, and human osteosarcoma MG-63 cell line, which derived from a 13-year-old male Caucasian patient. These cells were used in Tissue Engineering studies that investigate the impact of bioactive glasses (BGs) on osteogenic differentiation, viability, and proliferation since they are frequently considered to be “osteoblast-like” (Czekanska et al., 2012; Wilkesmann et al., 2020). HSF and MG-63 cells were cultured on the prepared scaffolds, and the viability of cells was measured 24 h after cell seeding. With viability percentages of approximately 99.0796%, 97.571 %, and 96.6991% for CNF-SBG, CNF-HA, and CNF-BBG, respectively, at low concentrations of 10 μg/mL, the results suggested that treatment of HSF with the scafolds with HA and BGs had a minor effect on the cells of HSF. Even at a high concentration of 100 ug/mL, no detectable cytotoxic effects were observed in HSF cells (95.7856%, 96.9067%, and 95.2874% for CNF-SBG, CNF-HA, and CNF-BBG, respectively, at concentrations of 100 μg/mL). Otherwise, the reverse microscope examination showed that none of the scaffolds had a high cytotoxic effect. Furthermore, MG-63 cells treated with CNF, CNF-HA, CNF-SBG, and CNF-BBG scaffolds significantly increased their viability up to Two times compared with the unmodified CNF membrane (Sabra et al., 2020; Samadian et al., 2020). This confirms the effectiveness of CNF fibres with bioceramic particles (HA and BGs) for periodontal tissue engineering applications (Elangomannan et al., 2017; Abd El-Aziz et al., 2021b).

### 3.3 Measurement of osteoblast differentiation and proliferation

**FIGURE 6:**
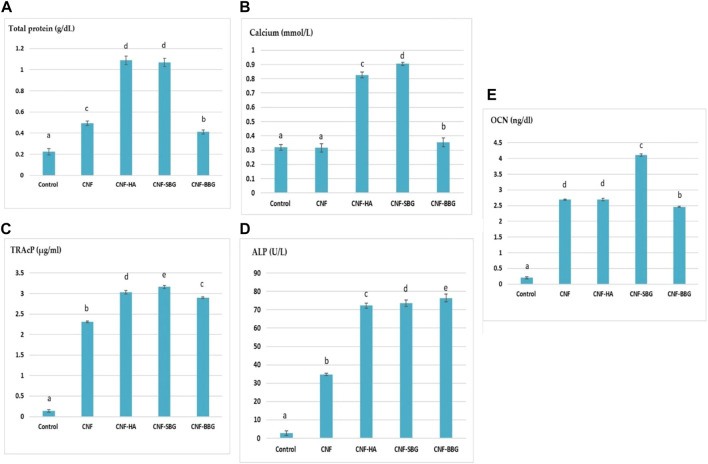
Effect of different scaffolds on levels of osteoblastic differentiation markers **(A–E)** of (total protein, calcium, TRAcP, ALP, and OCN) respectively.

The osteoinductive activity of the scaffolds is vital for repairing bone defects. So Osteoclast differentiation was studied by estimating the tartrate-resistant acid phosphatase (TRAP) and alkaline phosphatase (ALP) activity in a cell culture supernatant Figures 6C, D), TRAP, and ALP are an important markers of osteoclast differentiation and maturation (Wang et al., 2018; Xia et al., 2019). The treatment of MG-63 cells with CNF, CNF-HA, CNF-SBG, and CNF-BBG scaffolds significantly increased TRAcP-5b and ALP activities compared to that of control (OB) cells at *p* > 0.05. The lowest TRAcP-5b and ALP levels were shown in the case of CNF fibre treatment, while the highest levels were showed in the CNF-SBG and CNF-BBG scaffold groups, respectively. From our results, we can suggest that the CNF-SBG, CNF-BBG, and CNF-HA scaffolds increased the cellular mineralization of MG-63 cells (Salerno A and Oliviero, 2012; Lim, 2022), and these results agree with Halling Linder, Cecilia et al. (2017), in which they suggest a potential role for both bone ALP and TRAP-5b in skeletal mineralization (Halling Linder et al., 2017).

Early research has shown that increased calcium ion concentrations promote osteogenic cell proliferation, division, and differentiation. When osteoblasts start secreting mineral matrix, this is a sign of maturation and is called mineralization.

As shown in Figure 6B, this study examined the calcium ion levels in MG-63 cells following treatment with CNF, CNF-HA, CNF-SBG, and CNF-BBG scaffolds. Total calcium levels were found to be significantly higher when bioceramics were present, particularly in CNF-SBG scaffolds, which had the highest level at *p* > 0.05. This could be explained by calcium ions released from hydroxyapatite and bio active glass, which will promote osteogenic differentiation, when compared to the CNF group and control (MG-63) cells (Yang et al., 2019). This result is consistent with the results of ALP activity because higher Ca dissolution rates encourage osteoblastic cell proliferation and differentiation while the smallest amount of Ca ion delays the development of ALP (Dos Santos Gomes et al., 2022).

Osteocalcin (OCN) is one of the most prevalent proteins in bone and is produced exclusively by osteoblast cells. Our results in Figure 6E show that the OCN protein was considerably higher in the CNF scaffolds decorated with HA and BGs compared with the control group. The highest level was shown in the case of CNF-SBG treatment at *p* > 0.05. Thus, the results suggest that the incorporation of both bio active glass and hydroxyapatite facilitates the osteogenic differentiation of the cells, and that glass and HA loading further improve mineralization ability (Qu et al., 2021).

The amount of viable cells in an osteoblast culture and their capacity to secrete ALP and matrix proteins are closely correlated with protein concentration (Medeiros et al., 2021). As can be seen from Figure 6A , HA, SBG, and BBG incorporation into the CNF scaffolds resulted in significantly higher total protein levels when compared to regular MG-63 cells (Shaban et al., 2021a; b). The scaffolds made of CNF-HA and CNF-SBG had the highest *p* value. This outcome is consistent with the results of both ALP activity and cell viability reported above.

## 4 Conclusion

Tissue engineering is regarded as an excellent alternative to traditional treatments for treating bone abnormalities. Bone tissue’s ECM is made up of collagen nanofibers reinforced by HA crystals. As a result, there has been a lot of interest in creating a composite material made of nanofibers, bioceramics, and bioactive glasses that share similar porosity and surface properties. The different prepared CNF mats are advantageous for osteoblast proliferation and improve the surface mineralization process *in vitro*. The CNF-HA, CNF-SBG, and CNF-BBG nanofibers enhanced proliferation and osteoblastic functions by increasing markers of bone formation: osteocalcin, alkaline phosphates, and calcium synthesis. The resorptive activity of MG-63 cells cultured with different CNF nanofibers was weaker than that of control MG-63 cells. Therefore, CNF-HA, CNF-SBG, and CNF-BBG are promising candidates for biomaterials applications and osteoporotic bone regeneration.

## Data Availability

The original contributions presented in the study are included in the article/supplementary material, further inquiries can be directed to the corresponding author.
